# Influence on Forgiveness, Character Strengths and Satisfaction with Life of a Short Mindfulness Intervention via a Spanish Smartphone Application

**DOI:** 10.3390/ijerph18020802

**Published:** 2021-01-19

**Authors:** Juan Pablo Pizarro-Ruiz, Nuria Ordóñez-Camblor, Mario Del-Líbano, María-Camino Escolar-LLamazares

**Affiliations:** 1 Educational Sciences Department, Faculty of Education, University of Burgos, 09001 Burgos, Spain; rjpizarro@ubu.es (J.P.P.-R.); mdlibano@ubu.es (M.D.-L.); 2 Health Sciences Department, Faculty of Health Sciences, University of Burgos, 09001 Burgos, Spain; cescolar@ubu.es

**Keywords:** mindfulness, positive affect, negative affect, forgiveness, character strengths, satisfaction with life, positive psychology

## Abstract

Mindfulness-based interventions (MBI) are a recognized effective psychological practice characterized by attention control, awareness, acceptance, non-reactivity, and non-judgmental thinking obtained through the practice of meditation. They have been shown to be useful in reducing stress and enhancing well-being in different contexts. In this research, the effectiveness of an MBI was evaluated on variables that can promote successful job performance such as mindfulness trait, positive and negative affect, forgiveness, personality strengths and satisfaction with life. The intervention was carried out through a smartphone application called “Aire Fresco” (Fresh Air) during 14 days in the middle of the quarantine produced by the Covid-19 pandemic. The study sample was composed of 164 Spanish people who were distributed in two groups: control group and experimental group, which were evaluated before and after the intervention. The MANCOVA performed showed an overall positive effect of the intervention on the variables evaluated. The different ANCOVAs carried out showed that the intervention was beneficial in increasing mindfulness trait, reducing negative affect or increasing life satisfaction, among others. Our study is, as far as we know, the first to demonstrate the effectiveness of a brief intervention in mindfulness conducted using a smartphone application in Spanish.

## 1. Introduction

Mindfulness is defined as “*paying attention in a particular way: on purpose, in the present moment, and without judgment*” ([1], p. 4). Mindfulness-based interventions (MBI) are a recognized effective psychological practice characterized by attention control, awareness, acceptance, non-reactivity, and non-judgmental thinking obtained through the practice of meditation [2]. In this research, we will evaluate the effectiveness of an MBI applied through a mobile application. We want to know if variables such as positive affect, forgiveness, character strengths or satisfaction with life improve thanks to the intervention in a context of confinement produced by the COVID-19.

In recent years, MBI have experienced a great boom and have shown their effectiveness in different contexts, especially in the field of health, both as complementary therapy to treatment and as therapy to improve well-being [3]. Recent studies point out the beneficial effects of mindfulness practice on mental health, reducing stress, anxiety-depressive symptoms and improving sleep quality, among others [4,5,6].

Consistent with these previous findings, in the current situation of pandemic, the practice of mindfulness can be beneficial to manage the stress and discomfort present in people, as well as to promote their well-being. During the early stages of the pandemic, many researchers reported the need to provide interventions to support the mental health of people affected by the COVID-19 crisis [7], highlighting the use of MBIs [8,9,10]. For example, Matiz et al. [7] found that mindfulness improved resilience and well-being during the COVID-19 confinement in Italy. Similarly, Jiménez et al. [11] studied the role of mindfulness practice as a protective factor in the confinement situation, by fostering emotional resilience. They found that self-compassion, which increased with mindfulness practice, was associated with better coexistence during confinement. Thus, the practice of mindfulness has been associated with greater resilience and, therefore, better coping strategies.

Recently the practice of mindfulness has spread to other areas such as organizations. In this context, mindfulness empowers the wisdom of leaders [12], improves employee well-being and job performance [13,14], and is fundamental to creating a climate of trust [15]. In the workplace, previous studies show that mindfulness is effective in managing work-related stress [16], reducing social anxiety and aggression [17], as well as to increase happiness in employees [14]. Similarly, mindfulness has been positively associated with a number of strengths that are important for successful job performance; such as emotional intelligence [18], self-compassion and tolerance of ambiguity [19], coping skills [16], and management self-efficacy [20]. Regarding the above, the scientific literature raises the importance of some psychological variables such as satisfaction with life, affects and various aspects of personality in promoting positive job performance [21].

Satisfaction with life is one of the individual factors that can have a significant impact on productivity. A relevant aspect of achieving high satisfaction with life is to foster employee job satisfaction, i.e., to promote satisfactory experiences in organizations [22]. Job satisfaction is a pleasurable or positive emotional state resulting from an appraisal of one’s job [23]. Several studies have found that mindfulness has a positive influence on job satisfaction, either by moderating its relationship with other variables such as health-promotion lifestyle [24], or by directly influencing it [25].

Mindfulness has also been associated in some studies with character strengths, a set of positive personality traits that are morally valued and associated with the good life [26]. Mindfulness alone seems to work better to increase psychological well-being at work, while the combination with character strengths seems to influence employees on a motivational level, which increases task performance [27]. On the other hand, mindfulness has been related to positive and negative affect [28]. Both people with a high degree of positive affect and those who practice mindfulness, are more often in a state of full concentration and engagement, and feel very energetic [29], which has also been previously related to job performance [30].

The intervention whose effectiveness has been analyzed in this research has been carried out using a smartphone application. Technological advances have made it possible to conduct interventions via mobile phones in a cost-effective and flexible manner and to adapt them to users’ needs [31]. Currently, it is estimated that there are more than 31,000 health-related applications and they have been used in many areas such as the promotion of healthy habits, the control of chronic diseases, the follow-up and control of vital signs and the monitoring of fitness, among others [32]. Mobile health (mHealth) is an emerging field that has created new opportunities [33]. It offers advantages such as constant availability, greater access, equity of resources, immediate support, anonymity, personalized content, lower cost and increased capacity and efficiency of the service, the suppression of geographical barriers to treatment, and the ability to involve traditionally hard-to-reach groups [34]. With the increase in the number of people with access to smartphones, the complexity and potential of health applications will grow and have an increasing impact on the population [32]. Despite this, there is little information available on the quality of such applications, beyond subjective reviews published in providers’ online markets. As a result, the selection of these applications is based more on their popularity than on objective information about their effectiveness [35].

For this reason, in recent years there has been a lot of research with the aim of verifying the effectiveness of mobile applications in the treatment of different psychological disorders, which has led to an increase in the use of mobile phones as a therapeutic tool [36,37]. Many of these applications are based on the theory of mindfulness [31]. In this sense, mindfulness applications have shown to decrease perceived stress, depressive symptomatology and to improve sleep quality [31,38,39,40]. Similarly, they have proven effective in improving emotional well-being, increasing satisfaction with life and promoting positive mental health [41]. Such is the efficacy of these mobile applications that some studies suggest that interventions using mindfulness-based applications may show more benefit than traditional interventions [31,42]. Thus, Morrison-Wylde et al. [42] compared the effectiveness of two mindfulness interventions, one traditional and the other using a mobile application, on job satisfaction and burnout in a sample of novice nurses. The results showed that the intervention with mobile application provided more benefits compared to the traditional intervention in nurses without symptoms of post-traumatic stress, showing more compassion and job satisfaction, and less burnout.

However, beyond the field of mental health in which studies focus on psychopathological variables and the reduction of symptoms, few studies focus on exploring the possible positive consequences of using them, such as increasing satisfaction with life or developing different character strengths that may encourage better job performance. In this context, the aim of this research is to evaluate the effects of a short MBI carried out through a smartphone application on different variables that have been related to positive job performance such as mindfulness trait, affects, forgiveness, character strengths, and satisfaction with life. It is, as far as we know, the first study that demonstrates the effectiveness of a smartphone mindfulness application in Spanish.

## 2. Materials and Methods

### 2.1. Participants

This study was carried out with 281 Spanish university students from the University of Burgos in the Social Education (Faculty of Education) and Nursing (Faculty of Health Sciences) degrees, who were randomly assigned to Control Group (CG) or Experimental Group (EG). From this initial sample we deleted people who did not complete some of the intervention sessions, those who had done regular mindfulness practice before or those who did not answer some of the pre-test or post-test questionnaires.

Thus, the final sample was composed of 164 Spanish people between 15 and 60 years old (M = 22.06, SD = 6.00). Of these, 89 (54.3%) belonged to the experimental condition and 75 (45.7%) to the control condition.

A non-probabilistic convenience sampling was used to select the sample. They were recruited via email and asked to participate voluntarily in a study on the influence of the use of some mobile phone apps during confinement.

The mean age of the CG was 23.33 years (SD = 8.00) and the mean age of the EG was 20.97 years (SD = 3.15). Regarding gender, the CG consisted of 54 women (72.0%) and 21 men (28.0%), and the EG of 82 women (92.1%) and 7 men (7.9%). Although there is a pronounced bias towards the female population, this proportion is similar to the university population in the field of studies analyzed. Furthermore, the OECD [43] indicators on higher education indicate that there are large gender differences according to the field of study. For example, the percentage of women in the area of education in the United Kingdom is 74%, in Ireland it is 68% and in Spain it is 81%, and with regard to the area of health and social services in the United Kingdom it is 79%, in Ireland it is 75% and in Spain it is 72%. Furthermore, in other recent studies on mindfulness, the gender distributions are similar to those in our research [3,44,45]. Finally, according to a recent review that analyzed the gender distribution of all published peer-reviewed trials on mindfulness [46], men accounted for less than 29% of nearly 10,000 participants.

### 2.2. Instruments

Table 1 shows a summary of the main information of each of the assessment scales used in the study.

#### 2.2.1. Five Facet Mindfulness Questionnaire (FFMQ)

FFMQ was created by Baer et al. [47] from the five most commonly used questionnaires to measure mindfulness trait. It consists of 39 items which are answered according to a 5-point Likert-type scale (1 = never o very rarely true, 5 = very often or always true) that are grouped into five factors: (1) *observe* (ability to perceive internal or external stimuli), (2) *describe* (label the experience through words), (3) *act with awareness* (explicit attention to one’s own present activities), (4) *non-judgment of the inner experience* (not establishing a value judgment in the face of perceived thoughts, sensations or emotions) and (5) *non-reactivity to the inner experience* (distancing and period of time without reaction to the experience). These factors are grouped into a general factor that contemplates the traits of mindfulness. The questionnaire has adequate psychometric guarantees of reliability and validity, and has good sensitivity to differences in mindfulness practice [3,44,51]. It has been validated in different languages and cultures, in countries such as Spain, France, the Netherlands, Germany, China, Norway and Chile [52]. Cronbach’s alpha in the pre-test and post-test for FFMQ were 0.87 and 0.89, respectively. The Cronbach alphas for each factor in the pre-test and post-test can be found in the diagonal of Table 2.

#### 2.2.2. Positive and Negative Affect Scale (PANAS)

PANAS [29] consists of 20 items: 10 items for “positive affect” and 10 items for “negative affect”, which are answered according to a 5-point Likert-type scale (1 = very slightly, 5 = extremely) to measure the degree to which the person has experienced that emotion during the last week. This questionnaire has been extremely useful in the field of psychopathology to establish differences between anxiety and depression, or to establish relationships between stress and physical health [53]. Cronbach’s Alpha showed good reliability for positive affect (pre-test = 0.87 and post-test = 0.89), and for negative affect (pre-test= 0.85 and post-test = 0.88), respectively.

#### 2.2.3. Heartland Forgiveness Scale (HFS)

HFS [48] comprises 18 items that assess dispositional forgiveness. It consists of three factors that assess (1) a person’s willingness to forgive himself (six items, a person’s ability to forgive himself for his mistakes and the things he has done wrong), (2) a person’s willingness to forgive others (six items, a person’s ability to forgive another for hurting or disappointing him), and (3) a person’s willingness to forgive negative situations beyond the person’s control (six items, a person’s ability to forgive negative events or situations that are beyond his control such as illness or natural disasters). These three factors are grouped into an overall factor called Total Forgiveness (total HFS). The items refer to statements about forgiveness that the participants have to answer according to their personal experiences on a 7-point Likert scale (1 = mostly false for me; to 7 = mostly true for me). It has shown good reliability and adequate convergent and discriminating validity, for example with other forgiveness measures or mental health [48,54]. Cronbach’s alpha in the pre-test and post-test for total HFS were 0.79 and 0.82, respectively. Cronbach’s alphas for each factor in the pre-test and post-test showed good reliability and they can be found in the diagonal of Table 2.

#### 2.2.4. Brief Strengths Scale (BSS)

BBS [49] consists of 12 items that are answered according to a 5-point Likert scale (1 = totally disagree, 5 = totally agree). The scale was created to measure a model of three character strengths in people with and without mental health problems: (1) *Temperance Strength* (4 items, describes people who persist in achieving goals and exhibit self-control), (2) *Intellectual Strength* (four items, measures a person’s curiosity and enthusiasm for creativity) and (3) *Interpersonal Strength* (four items, describes a person’s love, concern and gratitude towards others). These three factors are grouped into an overall factor called Total Strengths (total BSS). This strengths model has been shown to be highly effective clinically in improving well-being, increasing resilience and reducing distress [49] and has been shown to be invariant with respect to gender, age, education levels and marital status [54]. Cronbach’s alpha in the pre-test and post-test for total BBS were 0.85 and 0.89, respectively. Cronbach’s alphas for each factor in the pre-test and post-test showed good reliability and they can be found in the diagonal of Table 2.

#### 2.2.5. Satisfaction with Life Scale (SWLS)

SWLS [55] consists of five items that are answered according to a 5-point Likert scale (1 = strongly disagree, 5 = strongly agree) and measures overall life satisfaction through the overall judgment people make about life. A higher score means higher satisfaction. It has been negatively related to negative affect, anxiety, depression and other psychopathological problems; and positively related to several measures of subjective well-being [55]. Cronbach’s alpha in the pre-test and post-test for total BBS were 0.83 and 0.83, respectively.

### 2.3. Procedure

A quantitative research approach with a quasi-experimental and longitudinal (pre-test and post-test) design with a non-equivalent control group was adopted in this study.

Participants were contacted via email asking for voluntary participation in a study on the influence of the use of certain mobile applications during the COVID-19 confinement. They were not given information on what the applications were and what they were to be used for. They were only informed that, depending on the group they belonged to, they would have to use one application or another, and that once the experiment started, they would not be able to exchange information about the applications they were using.

They answered the pre-test questionnaires between 15 and 16 April 2020 through an online survey platform (https://www.onlineencuesta.com). It should be noted that Spain was under strict house arrest (quarantine) from 14 March to 2 May 2020 and was not allowed to leave home except for reasons of force majeure. In other words, the participants had been under strict house arrest for one month when they answered the pre-test. Subsequently, the subjects were randomly assigned to the CG and EG. Both groups received an email with instructions to be followed during the next 14 days.

The EG was instructed to download and use a commercially available smartphone application (Aire Fresco (Fresh Air); https://www.airefresco.co) that would provide them with guided mindfulness sessions. The e-mail also explained that they should do one session per day for the next 14 days, i.e., from 17 to 30 April 2020. As in previous studies of MBI with mobile applications [45,55], the subjects did not receive any previous training in mindfulness, they were only given access to the application and instructed on its installation and functioning. The sessions lasted a mean of 15 min and 47 s, the longest being 26 min and 8 s, and the shortest 11 min and 47 s. This type of application has already been used effectively in recent research [45,55,56,57,58], but as far as we know, this would be the first study that would demonstrate the effectiveness of a mindfulness smartphone application in Spanish. Thanks to the developers of the application we obtained information about the follow-up of the sessions, and we were able to confirm that the participants adhered to the intervention plan. The participants were informed in advance and gave their consent for us to obtain access to their usage data before starting the study.

The CG received similar instructions, but in this case, participants had to download another application for smartphones (Lumosity, https://www.lumosity.com/es). This application proposes a series of activities popularly known as “mind training”. It offers games for attention, memory, speed, flexibility and problem solving. This application has also been used for CG in recent research on mindfulness [45,55,56,57,58,59]. Participants had to use this application for approximately 16 min per day on the same dates as the EG, i.e., between 17 and 30 April 2020.

After the 14 days of intervention, the EG and the CG answered the questionnaires of the post-test on the same online survey platform as in the pre-test. They did so between 1 and 2 May 2020.

### 2.4. Data Analyses

First, the correlations between the dependent variables of the study in the pre-test and in the post-test were calculated. Second, to check whether the two groups (CG and GE) were equivalent, the analysis of variance (ANOVA) of the pre-test scores was applied for all the dependent variables of the study (factors included): FFMQ, PANAS, HFS, BSS and SWLS. Then, to check the effectiveness of the intervention in mindfulness, analyses of the covariance (ANCOVAs) of the change scores (ChS = post-test scores minus pre-test scores) obtained by the EG and GC in the dependent variables were carried out, controlling the scores obtained in the pre-test. Finally, a MANCOVA was carried out to check the overall effectiveness of the mindfulness intervention on the dependent variables, controlling the pre-test scores.

In all analyses the effect sizes were estimated through the partial eta squared statistic (*ƞ_p_^2^*). It is considered that *ƞ_p_^2^* around 0.01 is a low effect, that a *ƞ_p_^2^* around 0.06 means a medium effect and that a *ƞ_p_^2^* above 0.14 is a large effect [60].

## 3. Results

First, Table 2 presents the internal consistencies and intercorrelations among study variables. Below the diagonal are shown the correlations between the variables in the pre-test, while above the diagonal are shown the correlations between the variables in the post-test. In both the pre-test and the post-test, the relationships between the variables were as expected. In particular, there were significant and positive relationships among many of the variables of positive nature such as FFMQ and positive affect, the dimensions of the forgiveness scale (except for the dimension others), the dimensions of the character strengths scale (except for the intellectual dimension) and satisfaction with life. Negative affect was in most cases significantly and negatively related to the rest of the variables.

Second, to test whether the CG and the EG were equivalent before the intervention, one-way ANOVAs were conducted with the pre-test scores of all dependent variables and using as independent variable (factor) the type of group (CG and EG) (see Table 3).

As can be seen in Table 3, there were statistically significant differences between the CG and EG participants in mindfulness trait (FFMQ), in FFMQ dimension five (non-reactivity of internal experience), and in temperance strength. On these three variables the CG scored higher than the EG. For the remaining variables, no significant differences were found between the two groups, revealing that the two groups were homogeneous in most of the dependent variables before the intervention began.

Next, to study if the intervention in mindfulness through the mobile application was effective, ANCOVAs were carried out with the ChSs obtained by the CG and EG in all the dependent variables, controlling the scores obtained in the pre-test using these scores as covariates (see Table 4). Although there were no significant differences between CG and EG in the pre-test variables, we decided to control them to avoid possible influences in the results.

As can be seen in Table 4, the EG experienced a greater change than the CG in most of the variables examined. The intervention was effective in increasing mindfulness trait (FFMQ) and its sub-dimensions observe, describe, act with awareness and non-judgment; in decreasing negative affect (PANAS); in increasing forgiveness as a trait (HFS) and its sub-dimension forgiveness towards oneself; in developing character strengths (BSS) and its sub-dimensions intellectual and interpersonal strength, and in increasing satisfaction with life. The effect sizes were mediums for mindfulness trait (FFMQ), non-judgment and satisfaction with life. For all other significant differences found, the effect sizes were small.

Finally, a MANCOVA was carried out (controlling the pre-test scores) to compare the CG and the EG, and to test the overall effectiveness of the mindfulness intervention on the dependent variables. The results showed significant differences between the two groups (Pillai’s Trace = 0.121, *F* = 3.45, *p* < 0.0001, *ƞ_p_^2^* = 0.161).

## 4. Discussion

The main objective of this research was to test the effectiveness of a short MBI carried out through a mobile application in Spanish. After 14 days of use, with only one daily session, participants improved in different variables related to successful job performance. Moreover, considering that people were in confinement because of the COVID-19 pandemic, we could conclude that this MBI is effective even in such circumstances.

Mindfulness interveners developed a greater ability to observe and describe their experiences, to act with greater awareness and attention to the present moment, and to not judge or make value judgments about their thoughts, emotions, or behaviors compared to CG individuals. It can therefore be concluded that the intervention was effective in increasing the mindfulness trait. It should be noted that this occurred even when in the pre-test the CG achieved significantly higher scores on the mindfulness trait than the EG. Previous literature suggests that the benefits of this increase in mindfulness would extend to both the professional and personal domains [61]. In a recent meta-analysis by Mesmer-Magnus et al. [61] of 270 studies (*n* = 58592) on the implications of mindfulness trait in the workplace, positive correlations were found with confidence, job satisfaction, job performance, mental health, emotional regulation, satisfaction with life and interpersonal relationships, while mindfulness trait was found to reduce burnout, perceived life stress, negative emotions, anxiety and depression. According to Glomb et al. [62] MBI has the power to improve self-regulation, making it easier to improve social relationships in the workplace, make employees more resilient to challenges and increase task performance.

As regards affect, our intervention failed in increasing positive affect scores, but it did decrease negative affect. The literature on the subject shows that negative affect is not simply the opposite of positive affect, but that they are separate and distinct dimensions, and that the same person can be both satisfied with his or her work and distressed [63]. People with high negative affect tend to emphasize the negative aspects of themselves and others, have lower thresholds for interpreting behavior as negative, and are more likely to be harassed at work, causing colleagues to identify them as weak and unable to defend themselves [64]. In addition, they avoid situations and activities that intimidate them because they believe they will not be able to cope successfully, experiencing increased stress and/or anxiety [65].

Thus, an intervention of this type in critical periods of stress or anxiety (such as the quarantine situation in which this research was carried out), means not only an improvement in the present moment, but also protection against future negative consequences. For example, Mikkelsen & Einarsen [66] showed that employees with higher levels of work-related distress tend to interpret subsequent events more negatively, predicting inadequate coping strategies, negative work attitude, emotional exhaustion and tension at work [64].

Regarding forgiveness, evidence suggests that forgiving oneself, forgiving others and negative events that are beyond a person’s control improves social relationships, self-esteem, cardiovascular health and immune function, and reduces anxiety, depression, stress and blood pressure [67]. Our MBI was able to improve overall forgiveness levels and those related to self-forgiveness, which were significantly higher in the EG than in the CG. However, there were no significant differences in forgiveness towards others or towards negative situations outside the person’s control. Mindfulness can help people in the forgiveness process in a number of ways: (1) increasing awareness that one’s self is in a state of unforgiveness, along with an understanding that this is causing harm or discomfort; (2) achieving better regulation of emotions through non-judgment or better understanding of the offender’s motivations through perspective, and (3) increasing forgiveness of oneself by making the person more open and curious, and less defensive [68].

The intervention was also effective in modifying character strengths, understood by positive psychologists as positive characteristics that manifest themselves in the thoughts, feelings and behaviors that enable people to thrive and that determine how the individual copes with adversity [49,69]. Our MBI improved intellectual and interpersonal strength. The former (intellectual) involves an increase in curiosity, enthusiasm and creativity that would be reflected in the greater vitality of the participants. It promotes diverse coping strategies in the individual by expanding and building new repertoires of thought and action [70]. The second (interpersonal) involves an increase in participants’ love, concern and gratitude towards others, which would be reflected in the maintenance of pleasant and satisfying relationships with other people [69]. It has also been associated with increased social support because individuals with this strength tend to have good social networks and more resources from which to obtain help, guidance and support in coping with stressful situations [71].

The intervention, conversely, did not result in the EG people scoring higher on the temperance strength than the CG people. However, it should be noted that the CG scored significantly higher than the EG in the pre-test, and these significant differences disappeared after the intervention. This temperance strength relates to self-regulation and the ability of individuals to persist in achieving goals and objectives [54]. Recent research has proposed to merge, in employees and organizations, on the one hand, character strength training interventions and, on the other hand, MBI, because of the demonstrated effectiveness of mindfulness on its own to increase work well-being and the strengths to influence at the motivational level and reinforce job performance [27].

Finally, the mindfulness intervention was also effective in increasing levels of satisfaction with life, which is consistent with previous studies that have found similar results (e.g., [72]).

Regarding the use of smartphone applications, as previously mentioned, there are several studies that find positive results in the users of this type of applications. Our results support the effectiveness of the “Aire Fresco” (Fresh Air) application in improving well-being and enhancing character strengths, like previous research. For example, Hubertly et al. [73] evaluated the use of a mobile application in reducing stress for 88 university students, finding significant differences in stress reduction, mindfulness and self-compassion. Bostock et al. [56] demonstrated the effectiveness of a mobile app in improving psychological well-being, distress, reduction of occupational stress and self- workday systolic blood pressure, and perceived social support during the working day. Sustained positive effects in the intervention group were found for well-being and job strain at the 16-week follow-up assessment. Some research even suggests the superiority of mindfulness interventions using smartphone applications over traditional intervention for certain types of people [42]. However, all this research has been conducted in English-speaking environments, with our intervention, to our knowledge, being the first to demonstrate the effectiveness of a Spanish-language smartphone app.

The results of this study should be interpreted in the light of some limitations. Firstly, the sample was largely composed of women, so the results should be explored in samples with greater representation of men. In addition, the effects of the intervention have not been evaluated in a post-test follow-up phase, so we have no data on whether the effects of the intervention are maintained over time. Future research should assess the long-term influence of the use of such mobile applications, similar to Bostock et al. [56]. It should also be noted that the participants were quarantined because of the COVID-19 pandemic, so it would be necessary to replicate the research in normal situations to see if the intervention through the mobile application maintains (or increases) its effectiveness. However, this has shown that mindfulness can be an effective technique for dealing with stressful life situations such as confinement. The intervention was carried out when the participants were in quarantine in Spain for 31 days, and during that time they were not allowed to leave their homes except for reasons of force majeure. This meant that people could not, for example, go for walks or play sports, unlike in other surrounding countries where they could. Finally, although our study explores the influence of intervention on variables related to work success, the sample was composed of university students. Future research should ensure that the results are similar using employees from different organizations.

## 5. Conclusions

In sum, this research has demonstrated the effectiveness of a short intervention in mindfulness through an application for smartphone in Spanish. Specifically, the intervention increased overall levels of mindfulness trait and most of its dimensions (i.e., observe, describe and act with awareness and without judgment), forgiveness in general and forgiveness towards oneself, as well as the levels of satisfaction with life. On the other hand, the intervention produced a decrease in the negative affect of the participants and influenced the increasing levels obtained in the intellectual and interpersonal strengths. Consequently, our study contributes to the accumulation of knowledge around mHealth as a valid orientation for health promotion.

Therefore, organizations should consider the usefulness of implementing short interventions in mindfulness through mobile applications such as “Aire Fresco” (Fresh Air), to promote the improvement of the occupational health of their employees and thus enhance their job performance.

## Figures and Tables

**Table 1:** Summary of the main characteristics of the instruments.

Name	Authors	Number of Items	Psychometric Properties
Five Facet Mindfulness Questionnaire (FFMQ)	Baer et al. [47]	Thirty-nine items which are answered according to a 5-point Likert-type scale	Cronbach’s alpha in the pre-test and post-test for FFMQ were 0.87 and 0.89, respectively
Positive and Negative Affect Scale (PANAS)	Watson, et al. [29]	Twenty items: 10 items for “positive affect” and 10 items for “negative affect”, which are answered according to a 5-point Likert-type scale	Cronbach’s Alpha showed good reliability for positive affect (pre-test = 0.87 and post-test = 0.89), and for negative affect (pre-test= 0.85 and post-test = 0.88), respectively.
Heartland Forgiveness Scale (HFS)	Thompson et al. [48]	Eighteen items that assess dispositional forgiveness	Cronbach’s alpha in the pre-test and post-test for total HFS were 0.79 and 0.82, respectively
Brief Strengths Scale (BSS)	Ho et al. [49]	Twelve items that are answered according to a 5-point Likert scale	Cronbach’s alpha in the pre-test and post-test for total BBS were 0.85 and 0.89, respectively
Satisfaction with Life Scale (SWLS)	Diener et al. [50]	Five items that are answered according to a 5-point Likert scale	Cronbach’s alpha in the pre-test and post-test for total BBS were 0.83 and 0.83, respectively.

**Table 2:** Internal consistencies, and intercorrelations among study variables (pre-test under the diagonal).

Variables	1	2	3	4	5	6	7	8	9	10	11	12	13	14	15	16	17
FFMQ	**0.87**/*0.89*	0.44 **	0.70 **	0.65 **	0.62 **	0.61 **	0.35 **	−0.43 **	0.54 **	0.50 **	0.23 **	0.52 **	0.34 **	0.34 **	0.14	0.36 **	0.42 **
Observe	0.40 **	**0.79**/*0.82*	0.25 **	−0.02	−0.13	0.40 **	0.12	0.10	0.11	0.09	0.10	0.08	0.30 **	0.118	0.21 **	0.40 **	0.02
Describe	0.66 **	0.20 **	**0.89**/*0.89*	0.32 **	0.18 *	0.38 **	0.27 **	−0.18 *	0.33 **	0.27 **	0.20 **	0.31 **	0.28 **	0.26 **	0.16 *	0.28 **	0.30 *
Act	0.68 **	0.08	0.27 **	**0.86**/*0.89*	0.42 **	0.11	0.23 **	−0.46 **	0.32 **	0.27 **	0.16 *	0.31 **	0.18 *	0.29 **	−0.01	0.16 *	0.31 **
Non-judgement	0.64 **	−0.19*	0.21 **	0.42 **	**0.91**/*0.92*	0.20 **	0.17*	−0.54 **	0.49 **	0.58 **	0.11	0.47 **	0.13	0.16 **	0.04	0.11	0.39 **
Non-reactivity	0.53 **	0.21 **	0.23 **	0.11	0.27 *	**0.87**/*0.89*	0.31 **	−0.08	0.33 **	0.24 **	0.13	0.41 **	0.13	0.18 *	0.01	0.14	0.20 *
Positive affect	0.35 **	0.19 *	0.17 *	0.29 **	0.17 *	0.21 **	**0.87**/*0.89*	−0.18 *	0.18 *	0.18 *	0.05	0.18 *	0.20 **	0.21 **	0.06	0.23 **	0.27 **
Negative affect	−0.37 **	0.17 *	−0.14	−0.38 **	−0.50 **	−0.14	−0.27 **	**0.85**/*0.88*	−0.41 **	−0.41 **	−0.15 *	−0.40 *	−0.09	−0.11	−0.04	−0.08	−0.38 **
HFS	0.53 **	0.12	0.28 **	0.30 **	0.50 **	0.31 **	0.30 **	−0.33	**0.79**/*0.82*	0.82 **	0.71 **	0.80 **	0.38 **	0.37 **	0.23 **	0.33 **	0.38 **
Oneself	0.55 **	0.03	0.35 **	0.31 **	0.57 **	0.26 **	0.25 **	−0.38 **	0.80 **	**0.73**/*0.71*	0.36 **	0.58 **	0.34 **	0.31 **	0.20 *	0.33 **	0.40 **
Others	0.09	0.10	0.04	0.05	0.04	0.01	0.04	0.01	0.31 **	0.19 **	**0.63**/*0.70*	0.30 **	0.27 **	0.20 *	0.31 **	0.15	0.19 *
Situations	0.52 **	0.15	0.20 **	0.29 **	0.47 **	0.41 **	0.36 **	−0.35 **	0.81 **	0.54 **	0.23 **	**0.73**/*0.72*	0.28 **	0.35 **	0.02	0.30 **	0.31 **
BSS	0.30 **	0.32 **	0.23 **	0.17 *	0.03	0.18 *	0.26 **	−0.04	0.40 *	0.30 **	0.27 **	0.33 **	**0.85**/*0.89*	0.80 **	0.82 **	0.85 **	0.38 **
Temperance	0.39 **	0.21 **	0.31 **	0.24 **	0.18 *	0.22 **	0.33 **	−0.10	0.43 **	0.34 **	0.18 *	0.42 **	0.78 **	**0.74**/*0.76*	0.49 **	0.52 **	0.41 **
Intellectual	0.10	0.22 **	0.07	0.09	−0.09	0.04	0.04	0.05	0.20 **	0.08	0.32 **	0.07	0.74 **	0.36 **	**0.76**/*0.87*	0.57 **	0.29 **
Interpersonal	0.20 **	0.32 **	0.15	0.07	−0.03	0.14	0.22 **	−0.02	0.30 **	0.25 **	0.15	0.26 **	0.83 **	0.44 **	0.47 **	**0.84**/*0.86*	0.25 **
SWLS	0.46 **	0.01	0.26 **	0.36 **	0.43 **	0.25 **	0.33 **	−0.34 **	0.42 **	0.43 **	0.06	0.42 **	0.41 **	0.43 **	0.17 *	0.34 **	**0.83**/*0.83*

Note. Cronbach’s are reported in the diagonal (**pre-test**/*pot-test*). ** *p* ≤ 0.01, * *p* ≤ 0.05. FFMQ: Five Facet Mindfulness Questionnaire; HFS: Heartland Forgiveness Scale; BSS: Brief Strengths Scale; SWLS: Satisfaction with Life Scale.

**Table 3:** Differences between CG and EG in the pre-test (one-way ANOVAs).

Dependent Variables	CG	EG	ANOVA		
	*n* = 75	*n* = 89			
	*M (SD)*	*M (SD)*	Pre-Test		
			*F*	*p*	*ƞ* ^2^
FFMQ	3.20 (0.38)	3.04 (0.42)	6.45	**0.012**	0.04
1. Observe	3.32 (0.68)	3.24 (0.68)	0.51	0.475	0.00
2. Describe	3.37 (0.68)	3.25 (0.72)	1.04	0.308	0.01
3. Act with awareness	2.99 (0.68)	2.87 (0.72)	1.29	0.258	0.01
4. Non-judgment of the inner experience	3.17 (0.93)	3.01 (0.79)	1.43	0.234	0.01
5. Non-reactivity to the inner experience	3.16 (0.47)	2.81 (0.51)	21.14	**0.000**	0.12
PANAS. Positive affect	2.03 (0.69)	1.83 (0.63)	3.76	0.054	0.02
PANAS. Negative affect	1.53 (0.79)	1.63 (0.72)	0.76	0.386	0.01
HFS	4.65 (0.71)	4.59 (0.73)	0.24	0.625	0.00
1. Forgiveness towards oneself	4.60 (0.99)	4.51 (1.04)	0.30	0.586	0.00
2. Forgiveness towards others	4.84 (1.02)	4.99 (0.80)	1.30	0.255	0.01
3. Forgive negative situations beyond the person’s control	4.50 (0.98)	4.26 (0.98)	2.49	0.117	0.02
BSS	4.23 (0.47)	4.10 (0.55)	2.55	0.112	0.02
1. Temperance strength	3.95 (0.64)	3.64 (0.69)	8.86	**0.003**	0.05
2. Intellectual strength	4.45 (0.51)	4.42 (0.62)	0.18	0.674	0.00
3. Interpersonal strength	4.28 (0.71)	4.25 (0.72)	0.10	0.748	0.00
SWLS	3.66 (0.71)	3.45 (0.72)	3.80	0.053	0.23

Note. M: Mean; SD: Standard Deviation; FFMQ: Five Facet Mindfulness Questionnaire; HFS: Heartland Forgiveness Scale; BSS: Brief Strengths Scale; SWLS: Satisfaction with Life Scale; In bold: *p* ≤ 0.05

**Table 4:** Differences between ChSs of the CG and EG by controlling the pre-test scores (ANCOVAs).

ChSs of the Dependent Variables	CG	EG	ANCOVA		
	*n* = 75	*n* = 89			
	*M (SD)*	*M (SD)*	ChS		
			*F*	*p*	*ƞ_p_^2^*
FFMQ	0.00 (0.29)	0.29 (0.42)	15.45	**0.000**	0.09
1. Observe	0.00 (0.51)	0.25 (0.66)	7.51	**0.007**	0.05
2. Describe	0.02 (0.49)	0.26 (0.55)	7.71	**0.006**	0.05
3. Act with awareness	0.04 (0.60)	0.30 (0.69)	4.86	**0.029**	0.03
4. Non-judgment of the inner experience	0.05 (0.63)	0.38 (0.62)	10.65	**0.001**	0.06
5. Non-reactivity to the inner experience	−0.12 (0.54)	0.24 (0.64)	1.61	0.207	0.01
PANAS. Positive affect	0.04 (0.55)	0.31 (0.74)	3.40	0.067	0.02
PANAS. Negative affect	−0.03 (0.56)	-0.36 (0.81)	8.06	**0.005**	0.05
HFS	0.08 (0.58)	0.31 (0.65)	5.81	**0.017**	0.04
1. Forgiveness towards oneself	0.09 (0.68)	0.38 (0.88)	5.65	**0.019**	0.03
2. Forgiveness towards others	0.05 (1.03)	0.14 (0.83)	1.82	0.179	0.01
3. Forgive negative situations beyond the person’s control	0.09 (0.99)	0.42 (0.97)	2.04	0.155	0.01
BSS	−0.12 (0.52)	0.08 (0.43)	5.10	**0.025**	0.03
1. Temperance strength	−0.05 (0.72)	0.14 (0.60)	0.24	0.623	0.00
2. Intellectual strength	−0.12 (0.59)	0.06 (0.55)	3.96	**0.048**	0.02
3. Interpersonal strength	−0.20 (0.62)	0.03 (0.58)	6.00	**0.015**	0.04
SWLS	−0.08 (0.57)	0.24 (0.54)	9.95	**0.002**	0.06

Note. M: Mean; SD: Standard Deviation; FFMQ: Five Facet Mindfulness Questionnaire; HFS: Heartland Forgiveness Scale; BSS: Brief Strengths Scale; SWLS: Satisfaction with Life Scale; In bold: *p* ≤ 0.05

## Data Availability

The data and protocols presented in the study are available from https://doi.org/10.17605/OSF.IO/XJEVS.

## References

[1] Kabat-Zinn J. (1994). Wherever You Go. There You Are: Mindfulness Meditation in Everyday Life.

[2] Bamber M.D., Morpeth E. (2019). Effects of mindfulness meditation on college student anxiety: A meta-analysis. Mindfulness (N. Y)..

[3] De la Fuente-Anuncibay R., González-Barbadillo Á., Ortega-Sánchez D., Pizarro-Ruiz J.P. (2020). Mindfulness and empathy: Mediating factors and gender differences in a Spanish sample. Front. Psychol..

[4] Strohmaier S. (2020). The relationship between doses of mindfulness-based programs and depression, anxiety, stress, and mindfulness: A dose-response meta-regression of randomized controlled trials. Mindfulness (N. Y)..

[5] Blanck P., Perleth S., Heidenreich T., Kröger P., Ditzen B., Bents H., Mander J. (2018). Effects of mindfulness exercises as stand-alone intervention on symptoms of anxiety and depression: Systematic review and meta-analysis. Behav. Res. Ther..

[6] Greeson J.M., Zarrin H., Smoski M.J., Brantley J.G., Lynch T.R., Webber D.M., Hall M.H., Suarez E.C., Wolever R.Q. (2018). Mindfulness meditation targets transdiagnostic symptoms implicated in stress-related disorders: Understanding relationships between changes in mindfulness, sleep quality, and physical symptoms. Evidence-Based Complement. Altern. Med..

[7] Matiz A., Fabbro F., Paschetto A., Cantone D., Paolone A.R., Crescentini C. (2020). Positive impact of mindfulness meditation on mental health of female teachers during the COVID-19 outbreak in Italy. Int. J. Environ. Res. Public Health.

[8] Behan C. (2020). The benefits of meditation and mindfulness practices during times of crisis such as COVID-19. Ir. J. Psychol. Med..

[9] Polizzi C., Lynn S.J., Perry A. (2020). Stress and coping in the time of COVID-19: Pathways to resilience and recovery. Clin. Neuropsychiatry.

[10] Zheng M.X., Yao J., Narayanan J. (2020). Mindfulness buffers the impact of COVID-19. Outbreak information on sleep duration. PsyArXiv.

[11] Jiménez Ó., Sánchez-Sánchez L.C., García-Montes J.M. (2020). Psychological impact of COVID-19 confinement andi its relationship with meditation. Int. J. Environ. Res. Public Health.

[12] Rooney D., Küpers W., Pauleen D., Zhuravleva E. (2019). A Developmental model for educating wise leaders: The role of mindfulness and habitus in creating time for embodying wisdom. J. Bus. Ethics.

[13] Reb J., Narayanan J., Chaturvedi S. (2014). Leading mindfully: Two studies on the influence of supervisor trait mindfulness on employee well-being and performance. Mindfulness (N. Y)..

[14] Coo C., Salanova M. (2018). Mindfulness can make you happy-and-productive: A mindfulness controlled trial and itseEffects on happiness, work engagement and performance. J. Happiness Stud..

[15] Hoy W.K., Gage C.Q., Tarter C.J. (2006). School mindfulness and faculty trust: Necessary conditions for each other?. Educ. Adm. Q..

[16] Zołnierczyk-Zreda D., Sanderson M., Bedyńska S. (2016). Mindfulness-based stress reduction for managers: A randomized controlled study. Occup. Med. (Chic. Ill).

[17] Clark L.B. (2020). Utilizing mindfulness based CBT to address anger and aggression in middle schools. J. Child Adolesc. Couns..

[18] Miao C., Humphrey R.H., Qian S. (2018). The relationship between emotional intelligence and trait mindfulness: A meta-analytic review. Pers. Individ. Dif..

[19] Fulton C.L. (2016). Mindfulness, self-compassion, and counselor characteristics and session variables. J. Ment. Heal. Couns..

[20] Becker B.D., Whitaker R.C. (2018). The association between dispositional mindfulness and management self-efficacy among early childhood education managers in head start. Mindfulness (N. Y)..

[21] Cequea M., Rodríguez Monroy C., Núñez Bottini M. Los factores humanos que inciden en la productividad y sus dimensiones. Proceedings of the 4th International Conference On Industrial Engineering and Industrial Management.

[22] De Oliveira M.Z., Natividade J.C., de Assis R.S., Barbosa Mambrini N.S. (2019). Performance, satisfaction and intention to remain in organizations: Individual to contextual predictors. Trends Psychol..

[23] Locke E.A. (1969). What is job satisfaction?. Organ. Behav. Hum. Perform..

[24] Lee J.H., Hwang J., Lee K.S. (2019). Job satisfaction and job-related stress among nurses: The moderating effect of mindfulness. Work.

[25] Choi J.I., Koh M.S. (2015). Relations of job stress, burnout, mindfulness and sob satisfaction of clinical nurses. Int. J. Bio-Science Bio-Technology.

[26] Peterson C., Seligman M.E.P. (2004). Character Strengths and Virtues: A Handbook and Classification.

[27] Pang D., Ruch W. (2019). Fusing character strengths and mindfulness interventions: Benefits for job satisfaction and performance. J. Occup. Health Psychol..

[28] Giluk T.L. (2009). Mindfulness, Big Five personality, and affect: A meta-analysis. Pers. Individ. Dif..

[29] Watson D., Clark L.A., Tellegen A. (1988). Development and validation of brief measures of positive and negative affect: The PANAS scales. J. Pers. Soc. Psychol..

[30] Lisbona A., Palaci F., Salanova M., Frese M. (2018). The effects of work engagement and self-efficacy on personal initiative and performance. Psicothema.

[31] Rung A.L., Oral E., Berghammer L., Peters E.S. (2020). Feasibility and acceptability of a mobile mindfulness meditation intervention among women: Intervention study. JMIR mHealth uHealth.

[32] Payne H.E., Lister C., West J.H., Bernhardt J.M. (2015). Behavioral functionality of mobile apps in health interventions: A systematic review of the literature. JMIR mHealth uHealth.

[33] Mani M., Kavanagh D.J., Hides L., Stoyanov S.R. (2015). Review and evaluation of mindfulness-based iPhone apps. JMIR mHealth uHealth.

[34] Grist R., Porter J., Stallard P. (2017). Mental health mobile apps for preadolescents and adolescents: A systematic review. J. Med. Internet Res..

[35] Stoyanov S.R., Hides L., Kavanagh D.J., Zelenko O., Tjondronegoro D., Mani M. (2015). Mobile app rating scale: A new tool for assessing the quality of health mobile apps. JMIR mHealth uHealth.

[36] Mohr D.C., Tomasino K.N., Lattie E.G., Palac H.L., Kwasny M.J., Weingardt K., Karr C.J., Kaiser S.M., Rossom R.C., Bardsley L.R. (2017). Intellicare: An eclectic, skills-based app suite for the treatment of depression and anxiety. J. Med. Internet Res..

[37] Cheung K., Ling W., Karr C.J., Weingardt K., Schueller S.M., Mohr D.C. (2018). Evaluation of a recommender app for apps for the treatment of depression and anxiety: An analysis of longitudinal user engagement. J. Am. Med. Informatics Assoc..

[38] Yang E., Schamber E., Meyer R.M.L., Gold J.I. (2018). Happier healers: Randomized controlled trial of mobile mindfulness for stress management. J. Altern. Complement. Med..

[39] Mistler L.A., Ben-Zeev D., Carpenter-Song E., Brunette M.F., Friedman M.J. (2017). Mobile mindfulness intervention on an acute psychiatric unit: Feasibility and acceptability study. JMIR Ment. Heal..

[40] Fish M.T., Saul A.D. (2019). The gamification of meditation: A randomized-controlled study of a prescribed mobile mindfulness meditation application in reducing college students’ depression. Simul. Gaming.

[41] Flett J.A.M., Hayne H., Riordan B.C., Thompson L.M., Conner T.S. (2019). Mobile mindfulness meditation: A randomised controlled trial of the effect of two popular apps on mental health. Mindfulness (N. Y)..

[42] Morrison Wylde C., Mahrer N.E., Meyer R.M.L., Gold J.I. (2017). Mindfulness for novice pediatric nurses: Smartphone application versus traditional intervention. J. Pediatr. Nurs..

[43] Ministerio de Educación, Cultura y Deporte, Instituto Nacional de Evaluación Educativa (2016). Panorama de la educación. Indicadores de la OCDE 2016. Informe Español.

[44] De la Fuente-Anuncibay R., González-Barbadillo Á., González-Bernal J., Cubo E., PizarroRuiz J.P. (2019). Mediating effect of mindfulness cognition on the development of empathy in a university context. PLoS ONE.

[45] Björkstrand J., Schiller D., Li J., Davidson P., Rosén J., Mårtensson J., Kirk U. (2019). The effect of mindfulness training on extinction retention. Sci. Rep..

[46] Bodenlos J.S., Strang K., Gray-Bauer R., Faherty A., Ashdown B.K. (2017). Male representation in randomized clinical trials of mindfulness-based therapies. Mindfulness (N. Y)..

[47] Baer R.A., Smith G.T., Hopkins J., Krietemeyer J., Toney L. (2006). Using self-report assessment methods to explore facets of mindfulness. Assessment.

[48] Thompson L.Y., Snyder C.R., Hoffman L., Michael S.T., Rasmussen H.N., Billings L.S., Heinze L., Neufeld J.E., Shorey H.S., Roberts J.C. (2005). Dispositionol forgiveness of self, others, and situations. J. Pers..

[49] Ho S.M.Y., Li W.L., Duan W., Siu B.P.Y., Yau S., Yeung G., Wong K. (2016). A brief strengths scale for individuals with mental health issues. Psychol. Assess..

[50] Diener E., Emmons R.A., Larsem R.J., Griffin S. (1985). The Satisfaction With Life Scale. J. Pers. Assess..

[51] Cebolla A., García-Palacios A., Soler J., Guillen V., Baños R., Botella C. (2012). Psychometric properties of the Spanish validation of the five facets of mindfulness questionnaire (FFMQ). Eur. J. Psychiatry.

[52] Rodríguez N.S. (2017). Mindfullnes: Instrumentos de evaluación. Una revisión bibliográfica. Revista Investig. Psicol. Soc..

[53] Sandín B., Chorot P., Lostao L., Joiner T.E., Santed M.A., Valiente R.M. (1999). Escalas PANAS de afecto positivo y negativo: Validación factorial y covergecia transcultural. Psicothema.

[54] Duan W., Ho S.M.Y. (2017). Three-dimensional model of strengths: Examination of invariance across gender, age, education levels, and marriage status. Community Ment. Health J..

[55] Bennike I.H., Wieghorst A., Kirk U. (2017). Online-based mindfulness training reduces behavioral markers of mind wandering. J. Cogn. Enhanc..

[56] Bostock S., Crosswell A.D., Prather A.A., Steptoe A. (2019). Mindfulness on-the-go: Effects of a mindfulness meditation app on work stress and well-being. J. Occup. Health Psychol..

[57] Economides M., Martman J., Bell M.J., Sanderson B. (2018). Improvements in stress, affect, and irritability following brief use of a mindfulness-based smartphone app: A randomized controlled trial. Mindfulness (N. Y)..

[58] Noone C., Hogan M.J. (2018). A randomised active-controlled trial to examine the effects of an online mindfulness intervention on executive control, critical thinking and key thinking dispositions in a university student sample. BMC Psychol..

[59] Lim D., Condon P., DeSteno D. (2015). Mindfulness and compassion: An examination of mechanism and scalability. PLoS ONE.

[60] Cohen J. (1992). Statistical power analysis. Curr. Dir. Psychol. Sci..

[61] Mesmer-Magnus J., Manapragada A., Viswesvaran C., Allen J.W. (2017). Trait mindfulness at work: A meta-analysis of the personal and professional correlates of trait mindfulness. Hum. Perform..

[62] Glomb T.M., Duffy M.K., Bono J.E., Yang T., Joshi A., Liao H., Martocchio J.J. (2011). Mindfulness at Work. Research in Personnel and Human Resources Management.

[63] Bang H., Reio T.G. (2017). PPersonal accomplishment, mentoring, and creative self-efficacy as predictors of creative work involvement: The moderating role of positive and negative affect. J. Psychol..

[64] Michel J.S., Newness K., Duniewicz K. (2016). How abusive supervision affects workplace deviance: A moderated-mediation examination of aggressiveness and work-related negative affect. J. Bus. Psychol..

[65] Samsudin E.Z., Isahak M., Rampal S., Rosnah I., Zakaria M.I. (2020). Individual antecedents of workplace victimisation: The role of negative affect, personality and self-esteem in junior doctors’ exposure to bullying at work. Int. J. Health Plann. Manage..

[66] Mikkelsen E.G., Einarsen S. (2002). Basic assumptions and symptoms of post-traumatic stress among victims of bullying at work. Eur. J. Work Organ. Psychol..

[67] Ogunyemi D., Sugiyama N.I., Ferrari T.M. (2020). A professional development workshop to facilitate self-forgiveness. J. Grad. Med. Educ..

[68] Braun S.S., Cho S., Colaianne B.A., Taylor C., Cullen M., Roeser R.W. (2020). Impacts of a mindfulness-based program on teachers’ forgiveness. Mindfulness (N. Y)..

[69] Yu L., Chan K.L. (2019). Moderating effects of personal strengths in the relationship between juvenile victimization and delinquent behaviors. Child Abus. Negl..

[70] Park N., Peterson C. (2009). Character Strengths: Research and practice. J. Coll. Character.

[71] Lavy S., Littman-Ovadia H. (2011). All you need is love? Strengths mediate the negative associations between attachment orientations and life satisfaction. Pers. Individ. Dif..

[72] Shankland R., Tessier D., Strub L., Gauchet A., Baeyens C. (2020). Improving mental health and well-being through informal mindfulness practices: An intervention study. Appl. Psychol. Heal. Well-Being.

[73] Huberty J., Green J., Glissmann C., Larkey L., Puzia M., Lee C. (2019). Efficacy of the mindfulness meditation mobile app “calm” to reduce stress among college students: Randomized controlled trial. JMIR mHealth uHealth.

